# Malaria in Palestine: the elephant in the room

**DOI:** 10.5281/zenodo.18336700

**Published:** 2026-01-22

**Authors:** Anton Alexander

**Affiliations:** 1 BC Business Centrum, Elscot House, Arcadia Avenue, London N3 2JU, United Kingdom

## Abstract

This paper examines the overlooked, forgotten or even ignored role played by malaria when considering the start of the Arab-Israel conflict in the Middle East. It would seem that anything associated with Zionism or Israel today is vilified, boycotted or shunned in certain quarters. Thus, by choosing to boycott anything that had a connection with Zionism or Israel, a study of the steps and events leading to the launch of the first start of sustainable malaria control has been missed, discouraging the malaria community of an opportunity to consider why and how certain methods were employed to achieve a desired successful outcome. In particular, there appears to be an attempt to suppress the fact that Palestine over 100 years ago was desolate, in many rural areas it was either almost empty or uninhabitable, due to the severity of the disease.

Palestine was described in 1919 as ‘one of the most highly malarious countries in the world’ by Dr. Manson-Bahr, later to become a Director of the London School of Hygiene and Tropical Medicine [1].

For many years, travellers had been accustomed to reading of Palestine’s desolation. Mark Twain, in 1867, wrote on his visit to Palestine:

*“A desolation is here that not even imagination can grace with the pomp of life and action…We never saw a human being on the whole route.”*

In its 1876 Handbook for Palestine and Syria, the travel agent Thomas Cook and Son said of Palestine that:

*“Above all other countries in the world, it is now a land of ruins. In Judea it is hardly an exaggeration to say that…for miles and miles there is no appearance of present life or habitation, …”*

Between 1882–1914, approximately 75,000 Eastern European Jewish idealists arrived to hopefully settle in Palestine (not to be confused with the religious Jews who for centuries came to try to live [and die] in the Holy Land). However, by 1914, about half this number of idealist Jews had died or had left, unable to cope with the severe pestilential conditions.

The question could be asked how a country as desolate or as sparsely settled as Palestine could have such a disease so widely spread and epidemics so continuous. The disease was spread throughout a number of Middle Eastern countries, the disease often attributed to movement of the roaming Bedouin. But Palestine, the Holy Land, attracted its own additional source of malaria through the constant active movement of those on annual pilgrimages. It was noted that this movement was as effective in spreading malaria and maintaining its endemicity as it would be in the case of any other infectious disease [2].

## World War I

In 1915, the year after the onset of WWI, the Turkish Army in Palestine attacked British positions on the Suez Canal, Egypt, but were repulsed by the British defenders. In 1916, the British Army invaded Palestine from Egypt, but attempts to capture Gaza in the south of Palestine were beaten back by the Turkish Army. In 1917, General Allenby was accordingly sent to replace the commander of the British Army then in Egypt. The British Army subsequently proceeded in 1917 with a successful invasion of Palestine as a conventional campaign, capturing Gaza and advancing northwards against the Turkish army with a view to occupying the southern half of Palestine.

## The Balfour Declaration

The British Foreign Secretary Arthur Balfour was an enthusiastic supporter of Zionism, and on 2^nd^ November 1917, the Balfour Declaration was issued in a letter from Balfour, the British Foreign Secretary, to Lord Rothschild:

*“His Majesty's Government view with favour the establishment in Palestine of a national home for the Jewish people, and will use their best end endeavours to facilitate the achievement of this object, it being clearly understood that nothing shall be done which may prejudice the civil and religious rights of existing non-Jewish communities in Palestine, or the rights and political status enjoyed by Jews in any other country.”*

It could possibly appear uncertain, perhaps even controversial, as to what was the British Government’s own agenda for the issue of the Declaration. The Government was aware of ‘the longest hatred’, antisemitism, and a popular initial explanation for the Declaration was that the British government had hoped the declaration would rally Jewish recruitment and opinion, especially in the United States, to the side of the Allied powers against the Central Powers (which included Germany and Turkey) during WWI. That goal may have been successful, but at the conclusion of the war, the intention of the Declaration may possibly have been to provide the British with a justification, an excuse, to remain in Palestine after the war and to claim Britain was trying to assist the Jews establish a national home.

Balfour had a definite interest for Britain to remain in Palestine. The Suez Canal had been opened in 1869. In 1875, as a result of debt and financial crisis, Egypt was forced to sell to the British government its shares in the company which operated the Suez Canal. The canal was crucial to British interests for its route to India and Middle Eastern oil. There was always a possibility that the British wished at some stage to control or administer Palestine. For many years before WW1, the British had discreetly shown a particularly keen interest in Palestine because it represented a buffer in the event it was necessary to protect their strategic colonial interests in, and the approaches to, the Suez Canal in neighbouring Egypt and thus ensure a vital communication route to British colonial possessions in India.

By now, in November 1917, thoughts of the Allied powers were already beginning to turn to who would be administering those parts of the Ottoman empire that were captured by the Allies. It was presumably then considered this would eventually be a decision for a body yet to be formed, the future League of Nations, and the choice of country would be made from those of the Allied powers willing to accept responsibility for administration.

It was likely there would be a competitive interest shown by the French who would go on to express an interest in administering Syria, Lebanon and Palestine. But the French interest may have been viewed more in the nature of empire building, whereas Balfour was presenting on behalf of Britain a specific project which was more in line with general ideals the to-be newly formed League of Nations would probably wish to encourage.

The Declaration had been drafted after previously informally consulting with the Allied Powers including France and the United States, presumably with a view to hopefully heading off later objections to Britain remaining in Palestine. Therefore, whilst Balfour genuinely wished to encourage Zionist ideas, the presence of Britain remaining in Palestine would be also consistent with trying to assist implementation of a Jewish national home, no matter how modest or small that might be, and Britain’s presence in Palestine would therefore discreetly provide protection for British Colonial interests after the war.

There appeared nothing controversial about the carefully worded Declaration. It seemed harmless, neutral, and it spoke specifically only of a homeland (not of a State nor of a government) in Palestine (not of the whole of, but only ‘in’ Palestine), and included a reference to the protection of non-Jewish rights, to emphasise prevention of harm to non-Jewish communities and ensuring no suggestion of a minority ruling a majority.

## The elephant in the room

The expression ‘elephant in the room’ is described as a major, obvious problem or controversial issue that everyone present is aware of but deliberately avoids discussing because it is uncomfortable, sensitive, or awkward.

It is here that a very significant aspect concerning the Declaration always seems to be overlooked or avoided by all who have examined or spoken about the Declaration. A Jewish national home would have required Jewish settlement in Palestine. But no-one could have foreseen, at that moment, the extent of the huge practical problems that would have had to be overcome for settlement in Palestine. Malaria, with its continuing existence in Palestine, had been known to the British Government to exist when the Declaration was drafted, but such Jewish settlement would have necessarily required **sustainable** malaria control for this to be achieved - yet such control did not then exist! Such malaria control was then completely unknown to the world’s scientific community. **Sustainable** malaria control had never before been seen or experienced anywhere in the world. Malaria control as a temporary measure was new, in its infancy, whilst a developmental stage of **sustained** control or of elimination was not yet even an idea. Malaria had rendered much of Palestine desolate, leaving it generally thinly populated and making it, in many rural areas, either uninhabitable or empty. Therefore, attempts at new significant settlement in Palestine by anyone (let alone by Jews – or even Arabs!) at that moment were unlikely to succeed, it may even have been perhaps impossible and potentially life threatening with prolonged exposure to the disease.

By December 1917, the British Army had advanced northwards from Egypt against the Turkish army and had occupied the southern half of Palestine. Allenby was then unexpectedly ordered to send some of his battle-ready troops to reinforce the British Army fighting in Europe, and instead he was sent troop replacements who required training. With the prospect of needing months to retrain these fresh troops, and finding his Army in such a malarious region, after personal inspection of the worst areas, Allenby ordered that anti-mosquito operations were to take precedence over all duties except prevention.

Accordingly, the British Army spent almost the next six months prior to 19^th^ September in the areas it occupied, thoroughly destroying the breeding sites of the anopheline mosquito near any British Army camp sites, and ensuring those breeding sites remained free of larvae. Such destruction of the breeding sites was a huge undertaking and was conducted under the direction of one of the best-known living authorities on mosquitoes, an experienced entomologist attached to the British Army, using over 60,000 men, mainly labourers of the Egyptian Labour Corps for this purpose [3]. Allen-by thereby effectively protected the bulk of his army from malaria whilst it retrained and regrouped for the final battle against the Turkish Army.

On the 19^th^ September, Allenby attacked. From the moment the British first crossed the front line from the ‘healthy’ British Army areas into the Turkish untreated positions, the British had a clear physical advantage by fighting a Turkish Army that was weakened through continuous exposure to malaria in the untreated Turkish positions.

It was already known that the incubation period for malaria was usually approximately 7-10 days after being bitten by an infected mosquito. Therefore, on 1^st^ October 1918, the severity of malaria in Palestine became suddenly and dramatically apparent – as if ‘the lights had gone out’ for the British Army. Having entered and been exposed to the disease in the ‘untreated’ conquered Turkish areas for just 11 days, from 19^th^ – 30^th^ September, from the 1^st^ October onwards for several weeks, more than 20,000 troops, over half of Allenby’s army, either died or collapsed from malaria, such was the severity of the disease. But by then, the Turkish Army had been defeated.

The Turkish Army had, in fact, between 19^th^ September and 1^st^ October been decisively defeated by a British Army incubating the malaria disease as it fought!

Of the captured Turkish troops, more than 20,000 of the 100,000 prisoners had to be admitted to hospital suffering from malaria.

All Allenby’s anti-mosquito work ceased on 19^th^ September, the work had served its purpose and mosquitoes quickly returned. Several years later, Allenby admitted he had taken a 10-day incubation period into account when planning the attack. He had in effect ‘weaponised’ malaria as his plan to totally defeat the Turkish Army within 10 days, and he was successful.

On 31^st^ December 1918, the Times newspaper printed a full-page detailed account by Allenby of the Turkish defeat that year in Palestine by his Army. Subsequent accounts of battles during WWI usually mention 19^th^ September 1918, the date when the British Army advanced from its position in the south of Palestine and attacked and decisively defeated the Turkish Army in the northern half of Palestine, and all accomplished within the following 11 days immediately after 19^th^ September. To the world, it was a great military victory and which was to be the final battle in the Middle East in WWI.

But the Times article only spoke of a historic military victory. This may have been the first seed sown that was to contribute to a future on-going Israel-Arab conflict because not once in the article was malaria mentioned. It was presented only as a brilliant military victory against the Turkish Army, without any recognition of either the necessity to control the disease (albeit temporarily during the 6 months), or of providing a physical advantage for the troops which enabled Allenby’s victory during those 10 days.

The omission was as if malaria had never existed.

A question could be asked, namely, was Allenby ordered not to mention the malaria and the extraordinary steps he took to overcome it? If it had become public knowledge that the malaria was so severe, Balfour’s suggestion of a national home might have appeared pointless, and any advantage he hoped to secure over any other country wishing to control Palestine by using the Declaration would have disappeared. It remains unknown why he omitted mentioning the malaria.

The steps taken to control malaria between March and September 1918 have never been told to the general public, and even today, these events are hardly known anywhere – not even by the malaria community. A Times reader could have reasonably concluded that Allenby had merely outmanoeuvred the Turkish Army, using conventional tactics and achieved a stunning victory.

But a comment in the official 1919 British Royal Army Medical Report [4] had noted:

*“It will probably be asked what would have happened to our troops had no antimalarial work been done, …The answer is that undoubtedly our troops would have simply melted away.”*

Allenby’s article had omitted to mention that without the antimalarial work, his army in all likelihood would have largely ceased to exist well before 19^th^ September.

Further, the Balfour Declaration’s encouragement of settlement to establish a national home was in effect ‘rubbished’, put down, or even suggested to be wholly impractical by a further note in the same 1919 British Army Medical Report:

*“It is interesting to speculate on what can be the future of a country such as [Palestine] from the health point of view. One cannot conceive the [malaria] problem [in Palestine] which faced the Army last spring [in 1918 during WWI] being undertaken by a Civil Authority. The expense alone would be prohibitive… The great bulk of the work [carried out by the British Army] was washed out by the first rains of October (1918).”* [4]

With statements such as these, it is not difficult to imagine the severity of the malaria that had savaged Palestine for centuries, and to also understand why Palestine was so desolate, uninhabitable in many areas. These 1919 statements were not widely publicised, and accordingly the general public would have been unaware there was a problem that would hinder or prevent settlement for a National Home.

Bluntly, the establishment of a National Home would have required settlement of a significant number of Jews, and, as at 2^nd^ November 1917, due to the severity of malaria, such settlement was probably neither physically nor practically possible and accordingly was then unrealistic.

## The Palin report

An event was subsequently to occur which may assist when considering the British motive for issuing the Declaration.

On 4^th^ April 1920 and for several days thereafter, a serious outbreak occurred in Jerusalem of Arab attacks on Jews in the course of which a number were killed and many wounded. Because there was still a British Military Administration in Palestine, a Military Court of Inquiry into the circumstances that gave rise to the violence was convened, with the Court comprising senior military officers, and with the conclusions of the court contained in the Palin Report [5].

The Report stated the Arabs viewed the Jews as:

*“…a race they have hitherto held in dislike and contempt’* (paragon. 6).

It continued (paragr. 7):

*“The Balfour Declaration … is undoubtedly the starting point of the whole trouble.”*

and (paragr. 8):

*“… by true meaning [of the Declaration], we must understand the only meaning intelligible to the population, in view of the loose references to the Declaration by the Allies orators and Press … that they were to be handed over to an oppression which they hated far more than the Turk’s and were aghast at the thought of this domination.”*

and (paragr. 9):

*“Rightly or wrongly, they fear the Jew as a ruler, regarding his race as one of the most intolerant known to history.”*

It is surprising that malaria was never mentioned in the report, nor was the severity of the disease, despite the following statement in the report in respect to which malaria would have had great relevance (parag.11):

*“The prospect of extensive Jewish immigration fills [the Arab] with a panic fear …’*

It is a puzzle why or how such anxiety had been allowed to exist in Palestine, particularly as all three of the Chief Administrators of Palestine and also Palin, the President of the Court, had been commanders in Allenby’s army and had witnessed the harm caused by malaria and may also have seen the British Army 1919 statement that had indicated any attempt at settlement was unrealistic.

The Balfour Declaration had been issued in November 1917, and was supposed to have been explained to the Palestine inhabitants, few as there were at that time. Apparently the Foreign Office pronouncement explaining the Declaration and Zionism (paragr. 32 of the Report) had been handed to three successive Chief military administrators in Palestine, each one declining the responsibility of publishing it. It was eventually published on 28^th^ April 1920 but this delay in publishing had left a vacuum. The Palin Report mentioned (paragr. 31) that the WWI Peace Negotiations with the Allied Powers had dragged on interminably, and without any indication from the British Administration during that period who would be administering Palestine after the military administration ceased. The Palin Report continued that this had invited every kind of speculation about the future administration of Palestine, and the Report states (paragr. 33) the inhabitants were ready prey for any form of agitation hostile to the British Government and to the Jews.

Major-General Palin had heard the evidence but allowed the Report to state that the Balfour Declaration was a settled matter, and (at paragr. 68) ‘it would most inevitably be executed’. It must be remembered that the British Army, earlier in 1919, had published a statement to the effect significant settlement was not possible. There was no further comment in the Report as to how it could possibly be achieved.

It would seem from the Report the principal matter that concerned the Arabs was of an incorrect belief they were about to be ruled or controlled by Jews. The fear of the arrival of significant Jewish settlement in Palestine was unrealistic if not impossible, and this never seemed to be mentioned.

The Report was typed and was then submitted to the British Foreign Office and War Office [6], who both agreed the Palin Report should not receive any publicity, it was concealed and noted as ‘secret’, treated as ‘confidential’, and was only eventually made available to the public in 1951, over 30 years later and after the British Mandate had ceased. The British Government had decided to suppress any publicity of the Report as it wished to avoid anything negative about the Balfour Declaration.

The three military Chief Administrators and Palin had been very much aware of the severity of the disease, and it would have been reasonable to assume they would have contradicted anything that arose which conflicted with their experience of malaria in Palestine. But that didn’t seem to be the case.

The 1920 Palin Report, the minutes and correspondence of the Foreign Office and of the War Office relating to the Palin Report, revealed a picture that has affected the Middle East to this day.

For the Balfour Declaration to be feasible, significant Jewish settlement in Palestine was required. It has already been mentioned that sustainable malaria control was unknown at that time. The British Army, in its 1919 Report, had already written that any realistic attempt to control malaria in Palestine was unlikely to be possible, and yet the British Government still chose to publicly promote and pursue its policy of achieving the (apparently then unachievable) objective of a Jewish National Home.

If the Balfour Declaration had been the only matter to be promoted, Britain would presumably have given up when significant settlement wasn’t deemed possible. But as will be seen, Britain didn’t give up, leading to an impression that the Declaration was useful as justifying Britain’s presence in Palestine to ostensibly assist with establishing a National Home.

Back in London, the Foreign Office and War Office strongly criticised the Military Administration [6] in Palestine for failing to administer the country according to British Government policy, which would have included correcting any alternative interpretations of the Balfour Declaration. The Secretary at the War Office wrote that the Balfour Declaration had been interpreted in a variety of ways by the people chiefly concerned. He continued that the Declaration was only fully understood in London by the Foreign Office and the War Office, but had never been understood in Palestine, except by the Zionists themselves.

The Foreign Office had even taken the step of appointing a Colonel to act as political officer to ensure the Military Administration in Palestine understood the significance of the Balfour Declaration, and with a view to stiffening the Administration.

It would appear the Balfour Declaration was an important card to play for the British Government in any negotiations as to who would administer Palestine. Control of Palestine by Britain was a very important matter, which seemed to be the situation even though significant Jewish settlement was probably unrealistic.

The bungling of the Military Administration was reflected in the Palin Report, illustrating matters that could and should have been either refuted or corrected by the Administration.

The Military Administration, by failing to comment or correct, had allowed the Declaration to be viewed as a Jewish takeover, which had incited the violence. (The principal agitator was named in the Report, Hagg Ameen el Husseini, but he had fled to escape British justice. Later, he was eventually to be granted an amnesty and he subsequently returned and became the Mufti, head of the Arabs in Palestine. His hatred of Jews, however, remained and due to his anti-British activities, he fled again in 1937 and spent WWII in Nazi Germany with Hitler, assisting with the Nazi war effort.

On 25 April 1920, the San Remo conference determined who would administer parts of the former Ottoman Empire and on 24^th^ July 1922, the League of Nations ratified the San Remo determinations of three League of Nations mandates: a French mandate for Syria, and British mandates for Mesopotamia and Palestine. With respect to Palestine, the resolution stated that the British were responsible *inter alia* for putting into effect the terms of the Balfour Declaration.

The Balfour Declaration has remained a symbol of a wrong for Arabs and anti-Zionists until this day. The whole situation seems to have been allowed to happen by a blundering Military Administration and the agitation appears to be based on a misconceived and incorrect suggestion that Britain gave Palestine away to the Jews.

The Balfour Declaration did however provide something positive. It provided encouragement, albeit misplaced, because attempts to then settle would have ended in failure and possibly even death. However, importantly, the Declaration certainly did stimulate a later search for ways to overcome the disease, to try to achieve ‘the impossible’. Centuries of antisemitism was a stimulus to persevere and in 1922, Palestine became the place for the eventual launch by the Zionists of the first start of successful sustainable malaria control.

The world was very sceptical of a successful outcome for this attempted malaria control, and in 1927, even the League of Nations issued a Report [7] to their Health Committee on the Work of the Malaria Commission:

*"... the sole purpose of the campaign against malaria, as far as the Commission is concerned, is to reduce the incidence and severity of the disease. Measures designed to accomplish more than that (particularly measures aiming at eradication) are not a wise proposition ... The Commission had occasion to enter into relations with a number of states which ... had undertaken an energetic campaign against malaria. The disease yielded to the measures which were taken, but reappeared with added virulence as soon as these measures were somewhat relaxed. This is a very serious result which brings in its train disillusion and discouragement.”*

But the Zionists ignored the 1927 Report and pressed on and in 1967, Israel was declared to be free of malaria. Otherwise rural Palestine could have remained as it was - desolate, uninhabitable and empty in many areas.

Without the encouragement provided by the Balfour Declaration, it is unknown what would have happened to the Zionist movement. If the Zionists hadn’t managed to develop sustainable malaria control, even with the Balfour Declaration, it is unlikely Israel would ever have come into existence.

The Balfour Declaration didn’t, couldn’t, have given Palestine away to anyone. Palestine was controlled by malaria, and whoever wrested control from malaria could have won Palestine.

It might be said malaria has always been the ‘elephant in the room’ in the Arab-Israel conflict.
